# Semi-field evaluation of the cumulative effects of a “Lethal House Lure” on malaria mosquito mortality

**DOI:** 10.1186/s12936-019-2936-2

**Published:** 2019-08-30

**Authors:** Antoine M. G. Barreaux, Welbeck A. Oumbouke, Innocent Zran Tia, N’guessan Brou, Alphonsine A. Koffi, Raphaël N’guessan, Matthew B. Thomas

**Affiliations:** 1 Department of Entomology, Center for Infectious Disease Dynamics, Pennsylvania State University, University Park, PA 16802 USA; 2 School of Biological Sciences, University of Bristol, Bristol, BS8 1TQ UK; 3 Institut Pierre Richet/Institut National de Santé Publique (INSP), Bouaké, Côte d’Ivoire; 4 London School of Hygiene and Tropical Medicine, Keppel Street, London, UK

**Keywords:** Vector control, Housing improvement, Mosquito entry, *Anopheles gambiae*, Screening, Lethal House Lure, Cumulative mortality, Feeding rate, EaveTube

## Abstract

**Background:**

There is growing interest in the potential to modify houses to target mosquitoes with insecticides or repellents as they search for human hosts. One version of this ‘Lethal House Lure’ approach is the In2Care^®^ EaveTube, which consists of a section of polyvinyl chloride (PVC) pipe fitted into a closed eave, with an insert comprising electrostatic netting treated with insecticide powder placed inside the tube. Preliminary evidence suggests that when combined with screening of doors and windows, there is a reduction in entry of mosquitoes and an increase in mortality. However, the rate of overnight mortality remains unclear. The current study used a field enclosure built around experimental huts to investigate the mortality of cohorts of mosquitoes over multiple nights.

**Methods:**

*Anopheles gambiae* sensu lato mosquitoes were collected from the field as larvae and reared through to adult. Three-to-five days old adult females were released inside an enclosure housing two modified West African style experimental huts at a field site in M’be, Côte d’Ivoire. Huts were either equipped with insecticide-treated tubes at eave height and had closed windows (treatment) or had open windows and open tubes (controls). The number of host-seeking mosquitoes entering the huts and cumulative mortality were monitored over 2 or 4 days.

**Results:**

Very few (0–0.4%) mosquitoes were able to enter huts fitted with insecticide-treated tubes and closed windows. In contrast, mosquitoes continually entered the control huts, with a cumulative mean of 50–80% over 2 to 4 days. Baseline mortality with control huts was approximately 2–4% per day, but the addition of insecticide-treated tubes increased mortality to around 25% per day. Overall cumulative mortality was estimated to be up to 87% over 4 days when huts were fitted with tubes.

**Conclusion:**

Only 20–25% of mosquitoes contacted insecticide-treated tubes or entered control huts in a given night. However, mosquitoes continue to host search over sequential nights, and this can lead to high cumulative mortality over 2 to 4 days. This mortality should contribute to community-level reduction in transmission assuming sufficient coverage of the intervention.

## Background

Many traditional African houses have open eaves and this gap between top of the wall and the roof is a key entry point for endophilic malaria vectors as they search for hosts to blood feed [1, 2]. A number of studies have demonstrated that closing or screening the eaves reduces the entry of malaria vectors [3–5] and in many contemporary houses (e.g., houses comprising brick walls and metal roofs), the eaves are often closed [6]. This sort of house improvement has been proposed as an important route to reducing disease burden [6–8]. In addition, there is growing interest in modifying houses to exploit mosquito host-searching behaviour and using the eaves for targeted delivery of insecticides [4, 9–12]. The WHO Vector Control Advisory Group (VCAG) characterizes this class of novel control tool involving housing improvement for targeted delivery of insecticides as a ‘Lethal House Lure’ (https://apps.who.int/iris/bitstream/handle/10665/274451/WHO-CDS-VCAG-2018.03-eng.pdf?ua=1). Placing insecticide-treated tubes within the eaves is one such approach [13–15].

The general concept involves sections of pipe (or possibly something like modified ventilation bricks) fitted into a closed eave, with some type of insecticide-treated netting placed inside the tube. The tube acts to channel human odour cues out of the house and as mosquitoes enter the tube, they contact the insecticide. A typical house might have 8–10 tubes and preliminary evidence suggests insecticide-treated tubes, combined with screening of doors and windows to make the house more ‘mosquito proof’, reduces entry of mosquitoes and increases overnight mortality rate, leading to reduced transmission risk at both household and community levels [14–17].

The epidemiological impact of the Lethal House Lure approach is currently being evaluated in a large-scale cluster randomized trial (CRT) in 40 villages in central Côte d’Ivoire [13]. Specifically, the CRT is testing the effect of household screening together with one version of a targeted insecticide delivery system: the In2Care^®^ EaveTube (see description below). In parallel with this CRT, small-scale studies are being conducted in Côte d’Ivoire to help better understand the functioning of EaveTubes, what the limitations are, and how the technology might be improved. Two recent studies [18, 19] have used field enclosures built around standard West African experimental huts [20, 21] to conduct release–recapture experiments to explore the impact of EaveTubes on overnight mortality of cohorts of mosquitoes. Both studies suggested that about 50% of mosquitoes that enter insecticide-treated tubes (in these cases, a wettable powder formulation of 10% beta-cyfluthrin) might die overnight, which is broadly in line with similar studies conducted in Kenya and Tanzania [15, 16]. Modelling analysis indicates that such levels of additional mortality should have a marked impact on malaria transmission [17]. However, the empirical data leave a number of unanswered questions. First, comparing across studies, it is slightly unclear what proportion of mosquitoes try to enter into the huts overnight and of those, what proportion contact eave tubes. Second, if mosquitoes do not seek to enter the hut/the tube one night, it is unclear whether they will be continually non-responsive, or whether they would go on to host seek in subsequent nights. Third, it is possible that some mosquitoes have very transient contact with insecticide and it is unclear whether they might suffer delayed mortality [22], or perhaps might receive insecticide exposures over multiple nights [23], neither of which would be apparent from a single overnight assessment.

The aim of the current study was to investigate these questions by introducing adult mosquitoes into a large enclosure containing two West African style experimental huts modified to contain insecticide-treated tubes at eave height and assessing how patterns of mortality change over 2 and 4 nights following release.

## Methods

### Set-up of the semi-field system

**Fig. 1:**
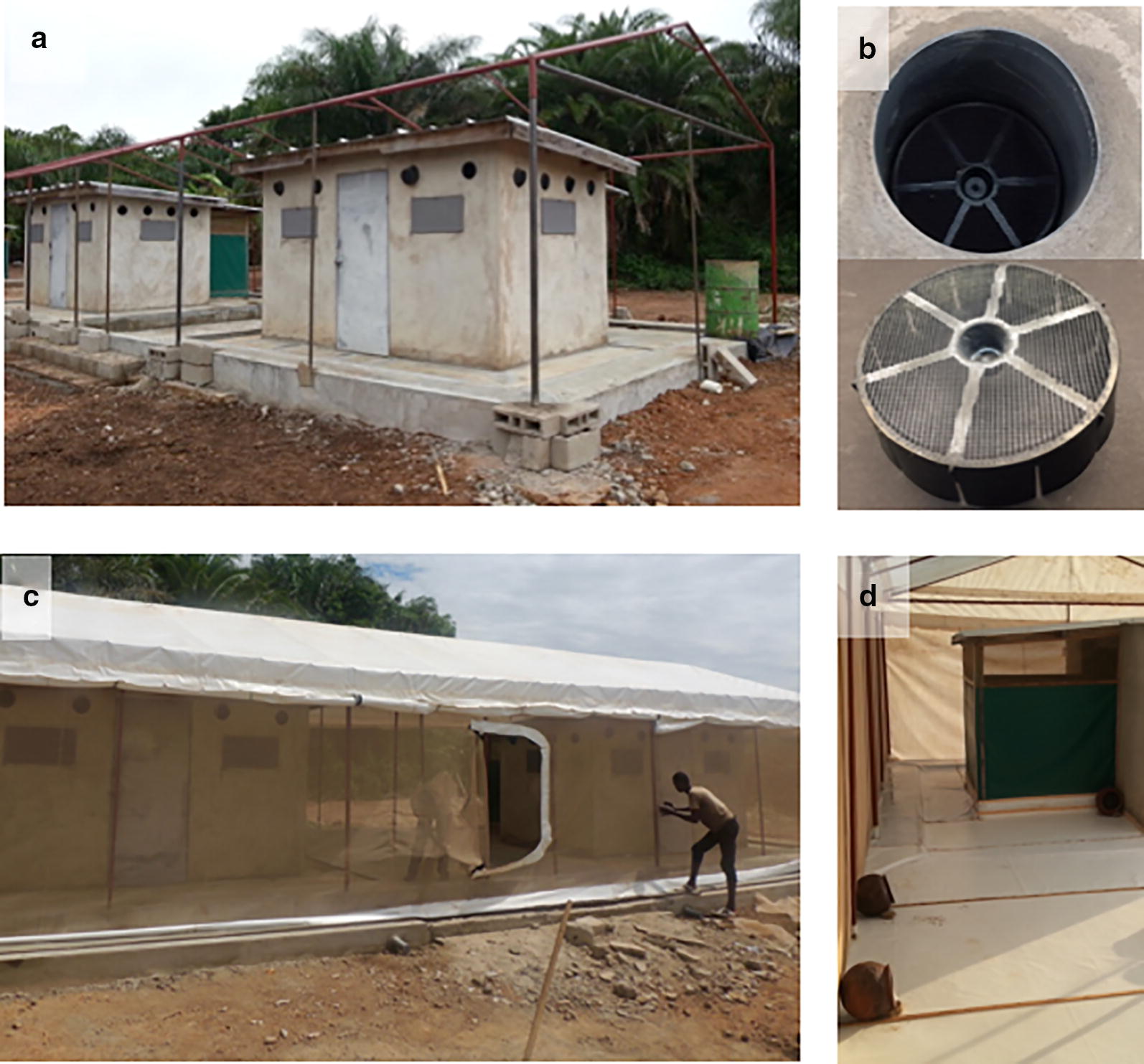
Semi-field enclosure for release–recapture studies. **a** Metallic framework of the enclosure around 2 modified West Africa experimental huts. **b** Top, insert inside an EaveTube (view from outside); bottom, treated insert with visible insecticide powder. **c** Netting walls and door, and tarpaulin roof. **d** White tarpaulin floor to facilitate collection of dead mosquitoes. Six clay pots (3 visible) containing 1 l water and cotton soaked in 10% sugar solution were installed in the enclosure the morning after the release to provide resting sites and sugar sources for mosquitoes

To conduct release–recapture studies, a screen house was erected to enclose two West African style experimental huts (Fig. 1) [18], at the experimental field site of M’be (5.209963 W and 7. 970241 N) [18, 24, 25]. EaveTubes were installed in the experimental huts [20, 21] by drilling 12–15-cm holes at eave level. A 20-cm long piece of PVC pipe was fixed inside each hole to house the EaveTube inserts. The drilling of 12 tubes per hut was actually for different experimental purposes. For the current experiments, only 6 holes (2 on each side of a hut and 2 at the front) were used and the remaining 6 holes were blocked. The holes were drilled at a 10° angle from the horizontal, pointing slightly upwards in the huts (Fig. 1) [18].

Six round clay pots (height 40 cm, diameter 30 cm) containing 1 l of water and also cotton soaked in 10% sugar solution (changed every day) were installed in the enclosure for each experimental run, one pot on each side of the veranda in the back of each hut (2 pots per hut) and 2 pots in the centre back of the enclosure (Fig. 1). These pots acted as resting sites and sugar sources for mosquitoes between nights. Temperature and humidity were recorded in the enclosure using a data logger placed just off the floor (on a brick) in the centre of the enclosure.

### Insecticide treatment

In the current experiment, insecticide was delivered in the tubes using plastic inserts holding a circle of netting treated with insecticide (produced by In2Care^®^, The Netherlands). The netting is treated with an electrostatic coating, which enables insecticide powders to bind to the netting. The inserts were machine-treated with a wettable powder formulation of 10% beta-cyfluthrin (Tempo 10©, Bayer) in the range of 300–500 mg of powder per insert [14], as used in the randomized controlled trial in Cote d’Ivoire. Mosquitoes entering the tubes are blocked by the inserts and upon contact insecticidal particles are transferred onto the mosquito body, delivering sufficiently high dose to potentially overcome insecticide resistance [14, 16, 19, 26].

### Mosquito populations

The study used *Anopheles gambiae* sensu lato (s.l.) mosquitoes derived from larval collections around M’be and Bouake in central Côte d’Ivoire [18]. These local populations are known to be highly resistant to pyrethroids [23, 27, 28]. Field-collected larvae were maintained at standard rearing density (about 300 larvae) in metallic bowls with 1 l of de-ionized water and fed daily with fish food (Tetramin™ baby) until pupation. Adult mosquitoes were housed in standard mosquito cages and maintained on 10% honey solution at 27 ± 2 °C, 60 ± 20% RH and ambient light.

### Mosquito release and recapture

Three-to-six days old, non-blood fed female mosquitoes were starved for 6 h before being released into the enclosure (sample sizes provided below). For recapture after release, mosquitoes were collected one-by-one inside the experimental huts and enclosure using a flashlight and individual glass haemolysis tubes plugged with a small piece of cotton. The position of each mosquito was recorded (i.e., whether inside one or other hut, or outside the huts in the enclosure), together with status (i.e., alive or dead). Mosquitoes were brought back to the laboratory at Institut Pierre Richet (IPR) research centre in Bouaké, Côte d’Ivoire, and identified to species level using a binocular microscope (40×).

### Experimental design

For each replicate, both huts inside the enclosure were assigned the same treatment: either (i) control, in which windows and tubes were open; or, (ii) treated, in which windows were closed and the tubes contained insecticide-treated inserts. Earlier studies demonstrated that there was no deflection between huts of different treatments [18] and so both huts were assigned the same treatment during a given release–recapture replicate. In all cases, a sleeper (human volunteer) was present in each hut, protected by an untreated bed net, and doors on the huts were closed.

- *Studies over two nights* Mosquitoes were introduced into the enclosure and monitored for 2 nights. For each introduction, 90–100 female *An. gambiae* s.l. were released in the central area of the enclosure at 20.15 h. The next morning at 05.00 mosquitoes that were inside the huts (dead or alive) or that were found dead on the floor of the enclosure were recovered. The second morning all remaining mosquitoes were recovered. The treatments and sleepers were rotated over the 2 huts with 20 total releases and 10 replicates of each hut treatment.
- *Studies over four nights* This experiment followed a similar protocol but was run over 4 nights instead of 2. For each introduction, 140–200 female *An. gambiae* s.l. were released in the central area of the enclosure at 20.15. Over the next 3 mornings at 05.00, mosquitoes found inside huts (dead or alive) or dead in the enclosure were recovered. The fourth morning, all mosquitoes were recovered. The treatments and sleepers were rotated over the 2 huts with 12 total releases and 6 replicates per hut treatment.

### Scavenging on dead mosquitoes by ants, and estimating mortality rate

The assessment of mosquito mortality in these experiments is based on collection of dead insects. However, in spite of using screening, a concrete floor and a water gutter around the enclosure, it was apparent that some ants could access the enclosure and potentially remove cadavers before they could be recovered. In order to estimate this removal rate, 250 freshly killed mosquitoes were distributed inside the enclosure at 8.15 p.m. and the number of cadavers remaining was assessed the following morning at 5.00 a.m. This experiment was replicated three times at approximately weekly intervals. The ‘average rate of removal of cadavers’ was used to estimate ‘maximum mortality’ from the ‘observed mortality’ based on collection of dead insects where

$$`{\text{Maximum mortality'}}\; = \;`{\text{Observed mortality'}}\; \times \;1/(1 - \;`{\text{Average rate of removal of cadavers'}}).$$

### Sample size calculations

The number of sample nights was above the number required to demonstrate 5% significance with 80% power. In first instance, the number of replicates was determined based only on the availability of mosquitoes, time and personnel. The replication was then checked retrospectively based on the empirical data using the “pwr pack- age” in R.

### Analysis

#### Mosquito entering huts

The cumulative proportion of mosquitoes entering huts for a given night is the total number of mosquitoes recaptured inside both huts up to that night divided by the number of mosquitoes initially released. These proportions were analysed using a linear mixed model that included hut treatment as an independent variable, and mosquito age at release a random effect.

#### Mosquito mortality

The cumulative proportion of dead mosquitoes for a given night is the total number of dead mosquitoes up to that night divided by the number of mosquitoes initially released. Mortality was analysed using a linear mixed model that included the hut treatment as an independent variable. Mosquito age at release was considered a random effect.

#### Maximum mortality taking scavenging into account

The maximum mortality, based on the estimated level of scavenging on cadavers and the observed mortality, was analysed using a linear mixed model that included the hut treatment as independent variable. Mosquito age at release was considered a random effect.

#### Linear mixed models

The differences in mosquito recapture and mortality rates between hut treatments were assessed using analysis of variance incorporating random effects (like mosquito age at release). The resulting linear mixed models were obtained in the software R version 3.5.0, using the lme4 package, version 1.1.15, and the “lmer” function.

Models were fitted and simplified for random effects using the likelihood ratio test (LRT). If a model with a given random effect was not significantly different from the same model without this random effect (P-value > 0.05) then the random effect was removed from the analysis. Models were compared using the “anova” function in the package lme4 and the maximum likelihood method (ML) [29–32].

The hut treatments (fixed effects) in the fitted linear mixed models were analysed using the restricted maximum likelihood (REML) approach (packages “lme4” and “lmerTest”) and the Kenward-Roger approximation [32–34]. Fixed effects with P-values > 0.05 were considered not significant.

## Results

Not all mosquitoes released into the enclosure were recovered. A mean ± SE of 73.4 ± 3.06% of the mosquitoes were recovered in the 2-day experiment (mean ± SE = 84.4 ± 2.24% when using control huts and mean ± SE = 63.5 ± 3.32% when using huts with eave tubes). In the 4-day experiment it was possible to recover a mean ± SE of 66.1 ± 5.45% of the mosquitoes (mean ± SE = 82.0 ± 2.91% in the controls and mean ± SE = 50.2 ± 4.59% in the eave tubes treatment).

Placing mosquito cadavers in the enclosures and assessing removal rate revealed substantial overnight scavenging on dead mosquitoes. The mean ± SE % removal of cadavers was 49.0 ± 2.00% per night. The ‘maximum mortality’ can now be estimated from the ‘observed mortality’ times 1.96 [i.e., 1/(1–0.49)].

### Mosquito hut entry

**Fig. 2:**
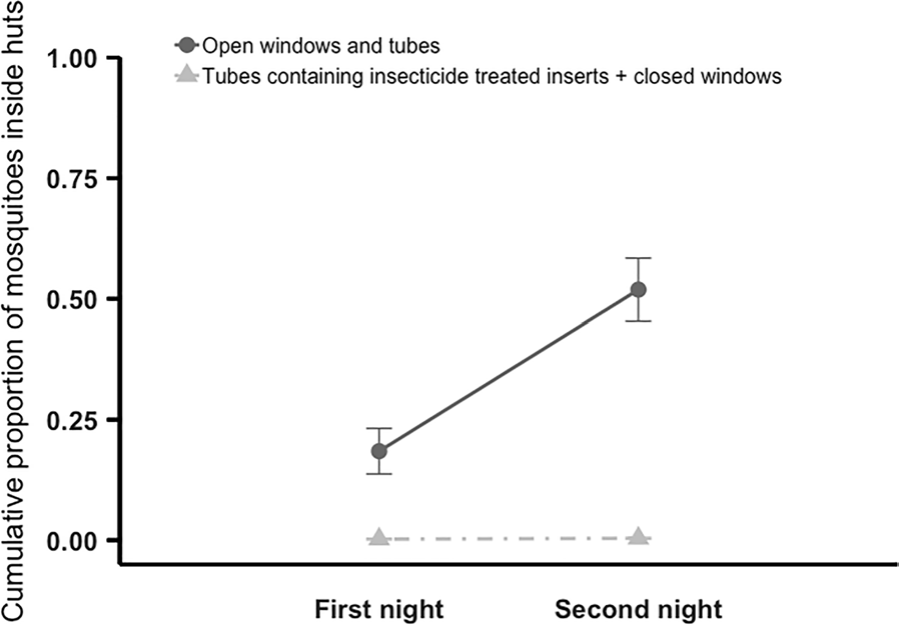
Cumulative mean (± SE) proportion of mosquitoes recovered inside huts within the enclosure over two nights. Adult *Anopheles gambiae* s.l. were released in the enclosure at 20:15. Mosquitoes found inside huts, or dead in the enclosure, were recovered at 05:00 each morning, over 2 nights. On the final morning all mosquitoes that could be found were recovered. Huts within the enclosure had either open windows and open tubes at eave height (control), or closed windows and tubes fitted with insecticide-treated inserts (treated). Over 2 nights mean ± SE of 0.4 ± 0.50% of mosquitoes entered treated huts and 51.9 ± 6.55% control huts. Means are based on 10 replicates of release–recapture per treatment

**Fig. 3:**
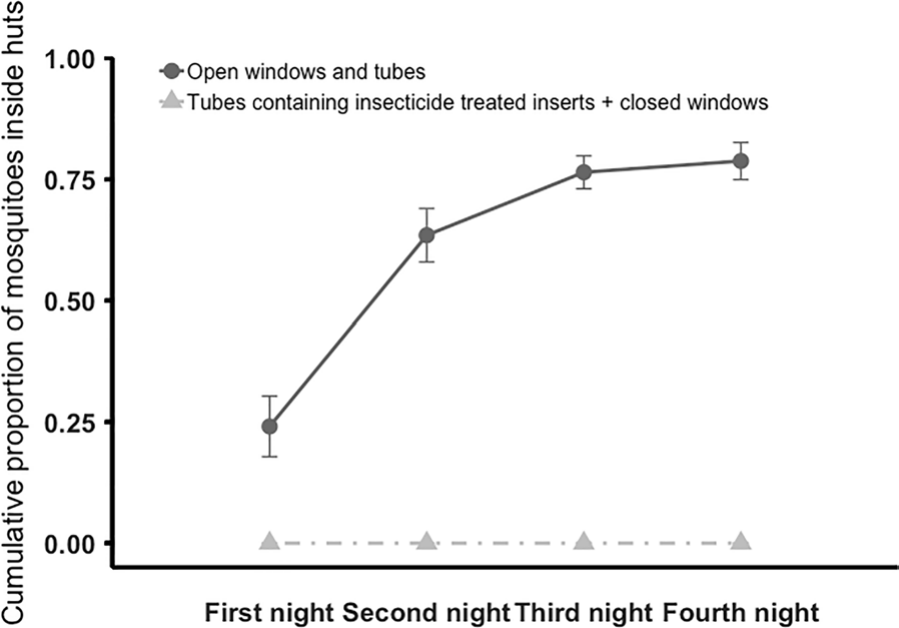
Cumulative mean (± SE) proportion of mosquitoes recovered inside huts within the enclosure over four nights. Adult *An. gambiae* s.l. were released in the enclosure at 20:15. Mosquitoes found inside huts, or dead in the enclosure, were recovered at 05:00 each morning, over 4 nights. On the final morning all the mosquitoes that could be found were recovered. Huts within the enclosure had either open windows and open tubes at eave height (control), or closed windows and tubes fitted with insecticide-treated inserts (treated). Over 2 nights mean ± SE of 0.0 ± 0.00% of mosquitoes entered treated huts and 78.8 ± 3.84% control huts. Means are based on 6 replicates of release–recapture per treatment

Hardly any mosquitoes were able to enter huts fitted with insecticide-treated tubes and closed windows (mean ± SE of 0.4 ± 0.50% in the two-night experiment, and 0.0 ± 0.00% in the four-night experiment) (Fig. 2). In contrast, mosquitoes continually entered the control huts, with a cumulative mean ± SE total of 51.9 ± 6.55% (F_1, 18_ = 66.48, P < 0.001) in the 2-day experiment (Fig. 2), and 78.8 ± 3.84% (F_1, 8_ = 392.17, P < 0.001) in the 4-day experiment (Fig. 3). The cumulative proportion of mosquitoes entering to huts appeared to reach a plateau about 4 days after release (Fig. 3). There was no effect of mosquito age (P > 0.05) in either study.

### Mosquito mortality

**Fig. 4:**
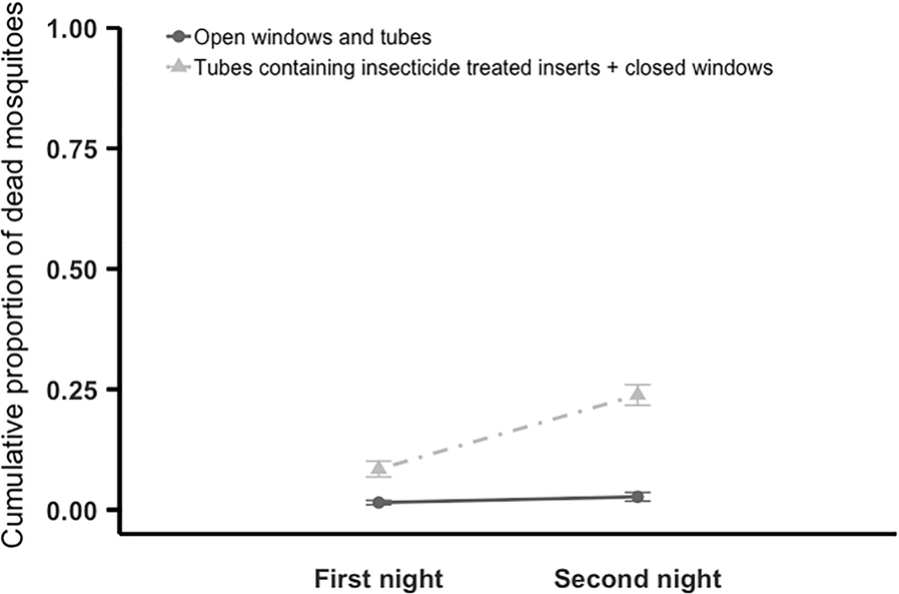
Cumulative mean (± SE) proportion of dead mosquitoes recovered inside the semi-field enclosure over two nights. Adult *An. gambiae* s.l. were released in the enclosure at 20:15. Mosquitoes found inside huts, or dead in the enclosure, were recovered at 05:00 each morning, over 2 nights. On the final morning all the mosquitoes that could be found were recovered. Huts within the enclosure had either open windows and open tubes at eave height (control), or closed windows and tubes fitted with insecticide-treated inserts (treated). Over 2 nights mean ± SE of 23.8 ± 2.14% of mosquitoes died when exposed to treated huts compared to 2.8 ± 1.00% with control huts. Means are based on 10 replicates of release–recapture per treatment

**Fig. 5:**
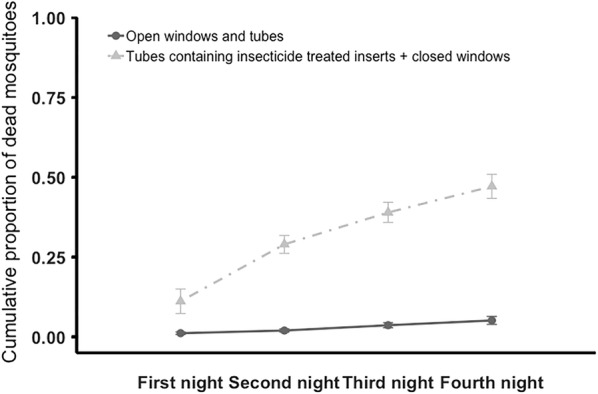
Cumulative mean (± SE) proportion of dead mosquitoes recovered inside the semi-field enclosure over four nights. Adult *An. gambiae* s.l. were released in the enclosure at 20:15. Mosquitoes found inside huts, or dead in the enclosure, were recovered at 05:00 each morning, over 4 nights. On the final morning all the mosquitoes that could be found were recovered. Huts within the enclosure had either open windows and open tubes at eave height (control), or closed windows and tubes fitted with insecticide-treated inserts (treated). Over 4 nights mean ± SE of 47.1 ± 3.77% of mosquitoes died when exposed to treated huts compared to 5.2 ± 1.25% with control huts. Means are based on 6 replicates of release–recapture per treatment

Significantly more dead mosquitoes were found when huts were equipped with insecticide-treated tubes compared to control huts with open eaves and open windows (Figs. 4, 5). In the 2-day experiment (Fig. 4), the cumulative mean ± SE % of dead mosquitoes was 23.8 ± 2.14% for the eave tube treatment but only = 2.8 ± 1.00% for the control (F_1, 11_ = 29.47, P < 0.001). In the 4-day experiment (Fig. 5) the cumulative mean ± SE  % of dead mosquitoes was 47.1 ± 3.77% with the huts fitted with insecticide-treated tubes compared to 5.2 ± 1.25% with control huts (F_1, 9_ = 120.19, P < 0.001). There was no effect of mosquito age (P > 0.05).

Correcting the number of cadavers recovered for the potential removal rate by scavengers suggests the maximum mortality could be as high as a mean ± SE of 46.5 ± 4.28% for the insecticide-treated tube treatment compared to 5.33 ± 1.95% in the control for the 2-day experiment (F_1, 18_ = 27.41, P < 0.001), and 86.7 ± 4.75% in the insecticide-treated tube treatment compared to 10.2 ± 2.36% (F_1, 9_ = 53.72, P < 0.001) in the control for the 4-day experiment. Again, there was no effect of mosquito age (P > 0.05).

## Discussion

The current study yields a number of results relevant to understanding the functional impact of insecticide-treated eave tubes. The continued host-seeking of mosquitoes over consecutive nights led to a cumulative total of 78.8% mosquitoes recovered inside the control huts over 4 days. The cumulative total for the 2-day experiments matched the day 2 total in the 4-day experiments very closely, indicating good repeatability of results. Very few mosquitoes were collected inside the huts fitted with insecticide-treated tubes. This result aligns with previous experiments [14, 18] and indicates that a well-screened structure can effectively block mosquito entry, providing ‘personal’ protection at the household level.

Baseline mortality (based on counts of cadavers) in the field cage set-up was approximately 2–4% per day. However, the addition of insecticide-treated tubes in the eaves turns the huts into a ‘lure and kill’ device [13, 15] and increases mortality to around 25% per day. This mortality is likely an underestimate as the cadaver removal experiment showed that in spite of efforts to prevent access of scavengers such as ants, around 50% of cadavers were removed from the field cage per night. If this removal rate is used as a correction factor, the estimated maximum cumulative mortality in the insecticide-treated tube treatment is approximately 47 or 87%, over 2 or 4 days, respectively. Interestingly, these corrected mortality levels closely match the cumulative percentage of mosquitoes recovered in the control huts and suggests that host-seeking is similar between treatments. The key difference, however, is that in the control huts the mosquitoes can enter through the open tubes/windows and are collected inside, whereas in the treated huts the mosquitoes are blocked from entering and contact with the insecticide-treated inserts leads to death outside the huts (and as the numbers match well between treatments it suggests that death occurs overnight, consistent with good contact with the inserts and little delayed mortality). This additional mortality should contribute to control at ‘community’ level [17].

Beyond the specific implications for EaveTubes, the study also provides some general insights potentially relevant to understanding feeding behaviour in (semi) field settings. Only a sub-set of mosquitoes appears successful in their host seeking in a given night. Given all mosquitoes were of similar age and feeding status (i.e., sugar starved and non-blood-fed) at the point of release, it is unclear why some mosquitoes took up to 4 days (or potentially longer) to enter the control huts or contact the eave tube inserts in the treated huts. It could be that all mosquitoes were equally responsive to host cues but that there is variation (biological or stochastic) in the ability of mosquitoes to find the limited entry points to the experimental huts. Alternatively, in spite of being the same physiological condition, there could be biological variation between mosquitoes in the motivation to blood feed. Whatever the mechanisms, this result has potentially important implications for understanding feeding frequency. A long-standing assumption used in most models of malaria transmission that biting rate can be approximated as the reciprocal of the duration of the gonotrophic cycle [35, 36]. In simple laboratory settings mosquito blood-feeding compliance is usually high and subsequent egg production relatively well synchronized, yielding little variation in duration of gonotrophic cycle between individual mosquitoes from the same environment [37–43]. However, in the current semi-field experiment, mosquitoes showed considerable variation in host seeking, which would in turn affect feeding frequency independent of the gonotrophic cycle.

One limitation of the study is that the recapture rate of mosquitoes (live or dead) was below 100%, with about 82% recapture rate for huts with open eaves and windows and 50–63% for huts with eave tubes and closed windows. The missing mosquitoes could have escaped from the enclosure, hidden themselves, been predated upon, or been scavenged. The high rate of scavenging estimated by examining removal of cadavers clearly indicates that the cage was not impermeable. More mosquitoes died when huts were equipped with insecticide-treated tubes, and the recapture rate was lower, so it seems plausible that scavenging by ants may be a factor of this lower recapture rate. The fact that not all mosquitoes could be accounted for adds some uncertainty to the absolute numbers reported here. However, it is not obvious why this should alter the interpretation of the relative treatment effects in terms of mosquitoes entering the huts or dying.

A further limitation is that the study used experimental huts rather than real houses and it is possible that mosquito entry rates and contact rates with insecticide, might differ between small experimental huts and real houses. These differences could result from differences in the structures [6, 21, 44, 45], complexity of the natural environment including availability of alternate blood and sugar sources [46–49], and in human behaviour [50, 51]. In addition, the current experiments compared treatments at the equivalent of 100% coverage (i.e., 2 control huts vs 2 treated huts). In reality, it is unlikely that coverage of an intervention would be implemented in every house within a given setting. As such, it is possible that the current experimental results overestimate the impact of EaveTubes and screening on mosquito mortality. Nonetheless, the effects are encouraging given evidence from modelling studies that indicate much lower coverage and lower mortality rates per feeding cycle can lead to marked impacts on malaria transmission potential [17].

## Conclusion

This study confirms that a well-screened structure can effectively block mosquito entry, providing personal protection at the household level. The addition of insecticide-treated tubes at eave height turns the house (in this case huts) into a Lethal House Lure and this additional mortality could contribute to control at the community level assuming high coverage of the intervention.

## Supplementary information

**Additional file 1.** Scavenging on dead mosquitoes. This data file gives the number of dead mosquito cadavers recaptured out of the 250 dead mosquito cadavers released the night before. It helps estimating the scavenging on dead mosquitoes by ants.

**Additional file 2.** Cumulative recapture over two nights. This data files gives the cumulative proportion of mosquitoes entering huts over two nights regarding the hut treatment and mosquito age.

**Additional file 3.** Cumulative recapture over four nights. This data files gives the cumulative proportion of mosquitoes entering huts over four nights regarding the hut treatment and mosquito age.

**Additional file 4.** Cumulative mortality over two nights. This data files gives the cumulative proportion of dead mosquitoes over two nights regarding the hut treatment and mosquito age.

**Additional file 5.** Cumulative mortality over four nights. This data files gives the cumulative proportion of dead mosquitoes over four nights regarding the hut treatment and mosquito age.

## Data Availability

All data generated or analysed during this study are included in this published article and in Additional files 1, 2, 3, 4, 5.

## Abbreviations

CRT: cluster randomized trial
IPR: Institut Pierre Richet
LRT: likelihood ratio test
ML: maximum likelihood
PVC: polyvinyl chloride
REML: restricted maximum likelihood
WHO: World Health Organization
